# ABrowse - a customizable next-generation genome browser framework

**DOI:** 10.1186/1471-2105-13-2

**Published:** 2012-01-05

**Authors:** Lei Kong, Jun Wang, Shuqi Zhao, Xiaocheng Gu, Jingchu Luo, Ge Gao

**Affiliations:** 1 College of Life Sciences, State Key Laboratory of Protein and Plant Gene Research, Center for Bioinformatics, Peking University, Beijing, 100871, P.R. China; 2 STG Lab Based Services, China Development Lab, IBM (China) Investment Co Limited, Beijing 100193, P.R. China

## Abstract

**Background:**

With the rapid growth of genome sequencing projects, genome browser is becoming indispensable, not only as a visualization system but also as an interactive platform to support open data access and collaborative work. Thus a customizable genome browser framework with rich functions and flexible configuration is needed to facilitate various genome research projects.

**Results:**

Based on next-generation web technologies, we have developed a general-purpose genome browser framework ABrowse which provides interactive browsing experience, open data access and collaborative work support. By supporting Google-map-like smooth navigation, ABrowse offers end users highly interactive browsing experience. To facilitate further data analysis, multiple data access approaches are supported for external platforms to retrieve data from ABrowse. To promote collaborative work, an online user-space is provided for end users to create, store and share comments, annotations and landmarks. For data providers, ABrowse is highly customizable and configurable. The framework provides a set of utilities to import annotation data conveniently. To build ABrowse on existing annotation databases, data providers could specify SQL statements according to database schema. And customized pages for detailed information display of annotation entries could be easily plugged in. For developers, new drawing strategies could be integrated into ABrowse for new types of annotation data. In addition, standard web service is provided for data retrieval remotely, providing underlying machine-oriented programming interface for open data access.

**Conclusions:**

ABrowse framework is valuable for end users, data providers and developers by providing rich user functions and flexible customization approaches. The source code is published under GNU Lesser General Public License v3.0 and is accessible at http://www.abrowse.org/. To demonstrate all the features of ABrowse, a live demo for *Arabidopsis thaliana *genome has been built at http://arabidopsis.cbi.edu.cn/.

## Background

With the rapid development of the next-generation sequencing technologies, more and more genomes of various species have been sequenced, bringing challenges for effective data management and analysis. By systematically integrating multiple heterogeneous annotations into a uniform interface, genome browser has greatly pushed forward the understanding of genomes. Nowadays, it has become an indispensable tool for both computational and bench biologists [1]. Given that building a full-functional browser from scratch is tedious and time consuming, a well-designed genome browser framework is even more important in the genomic era.

The generic genome browser (GBrowse) [2] offers a portable framework for genome demonstration, and has been widely used for several model organism genome projects such as TAIR [3], FlyBase [4] and WormBase [5]. With all source codes publically available, Ensembl [6] and UCSC genome browser [7] can also serve as browser framework for customizing genome demonstration, e.g., Gramene [8] and Vega [9].

Online browsing is the main approach to access data in a genome browser. By providing graphic view for the multiple heterogeneous annotation data, a genome browser allows researchers to visually analyze interesting entries and inspire novel discoveries. However, the static page-based implementation used by classical genome browsers results in discontinuous page transitions and disruption of user attention, especially during navigation through large genomic regions with multiple annotations [10]. By employing the AJAX-based web technology, some new genome browsers such as JBrowse [10], Anno-J [11] and Genome Projector [12] overcome this deficiency, enabling smooth navigation and improving user experience significantly.

Besides graphical data browsing, integration with external applications is also valuable to facilitate further data analysis [13]. Based on Galaxy [14] and GREAT [15] standard interfaces, UCSC genome browser [16] supports users to submit selected data by simple clicks, connecting the annotation data resource with computation tools transparently.

In addition to human-oriented interface, machine-oriented data retrieval is becoming even more essential for large-scale data analysis [13]. Therefore, several well-defined protocols have been exploited to openly access the rich resources in genome databases, in order to help integrate multiple online resources into workflows [17] for comprehensive data analysis. Web service [18,19] is well designed for this purpose and has been widely used for exchanging structured information through networks. With the built-in BioMart [20,21] system, Ensembl supports standard SOAP-based web service API. Moreover, GBrowse [2], Ensembl [22] and UCSC genome browser [7] allow external programs to access pre-compiled annotations via BioDAS [23], a dedicated communication protocol for exchanging biological annotations.

The rapid increase of massive heterogeneous genomic data puts great demands on close collaborations among various researchers with diverse backgrounds. Sharing annotations and comments among individual researchers contributes valuable information to the community, and will significantly accelerate novel discoveries [18,24]. Therefore, most of the popular genome browsers allow users to upload and display their own annotations as custom tracks, and Ensembl also supports users to add comments to existing annotation records.

Built upon cutting-edge web technologies, ABrowse provides a general-purpose framework for visualizing and analyzing large-scale heterogeneous genomic data. For end users, ABrowse offers a map-like web interface for navigating and annotating the whole genome in a highly interactive manner. Through various standard data access interfaces, users can easily access abundant annotations from back-end genome databases, and further integrate the data into their own analysis workflows. User-generated contents (UGCs) can be interactively added and seamlessly integrated with existing annotations on-the-fly. Moreover, all UGCs can be freely shared with colleagues or kept as private for the contributor. For the data provider and site administrator, ABrowse provides several administration utilities for loading new annotation tracks conveniently and customizing page appearance. Furthermore, ABrowse also provides open APIs for developers to write new plug-ins and fine-tune behaviors to meet their own requirements.

Released as free software under GNU Lesser General Public License v3.0, all source codes of ABrowse can be downloaded freely online http://www.abrowse.org/. Detailed documents are provided for end users, site administrators and developers. A live demo for *Arabidopsis thaliana *genome http://arabidopsis.cbi.edu.cn/ is built to demonstrate all the features of ABrowse.

### Implementation

**Figure 1:**
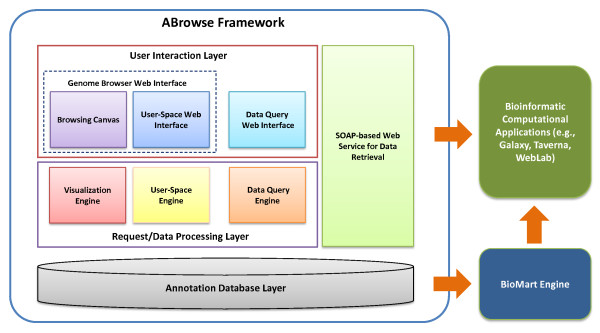
**ABrowse architecture**. ABrowse framework contains three layers: the user interaction layer, the request/data processing layer and the database layer. Moreover, the standard web service is provided for open data access. The default database scheme is compatible with BioMart, thus developers can configure a BioMart instance to access data stored in ABrowse databases. All the data stored in ABrowse system can be openly accessed from external bioinformatic computational applications seamlessly for further analysis.

As shown in the ABrowse architecture design chart (Figure 1), there are three layers in ABrowse framework: the user interaction layer, the request/data processing layer and the annotation database layer. The user interaction layer consists of the genome browser web interface with a built-in user-space, and a data query web interface. The request/data processing layer contains engines for visualization, user-space management and data query to process user requests. The annotation database layer stores and manages genome annotation data from both the built-in system and external community users. Besides these three layers, a SOAP-based web service for remote data retrieval is built on top of the annotation database layer for open data access with programming interface.

To support interactive user experience, the genome browser web interface is implemented in JavaScript framework powered by ExtJS http://www.sencha.com/products/extjs/. At the server side, the visualization engine is implemented in Java and designed in strategy pattern, helping users to plug in their own drawing strategies easily. In addition, ABrowse employs the Lucene search engine http://lucene.apache.org/ to provide full text search function for the underlying annotation data.

Massive amounts of data bring challenges for data organization and retrieval. In order to handle the flood of next-generation sequencing data efficiently, ABrowse employs MySQL spatial database index http://dev.mysql.com/doc/refman/5.0/en/spatial-extensions.html for back-end data storage by default, helping to simplify query statement and increase query speed (Additional file 1, Figure S1). Furthermore, ABrowse also supports different database management systems for the back-end database to meet various user requirements.

Since the default database scheme of ABrowse is compatible with BioMart [20,21], developers can easily configure a BioMart instance for data retrieval. In addition, all the data stored in ABrowse can be openly accessed by external bioinformatic computational applications for further analysis.

## Results and Discussion

### Interactive Web Interface

With the development of new web technologies, rich internet application enables users to interact with the application without having to wait for the server. Powered by cutting-edge web technologies, ABrowse offers end users highly interactive browsing experience by supporting smooth genomic feature navigation.

**Figure 2:**
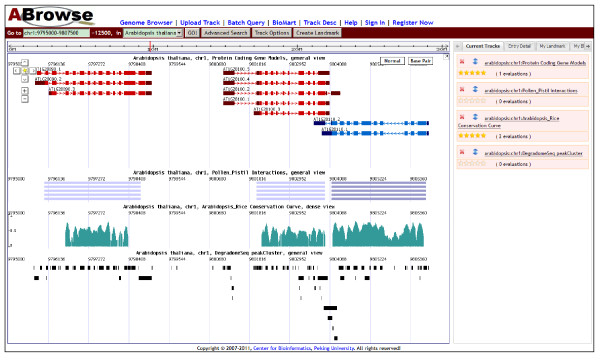
**The main interface of ABrowse genome browser**. The genome browser web interface mainly consists of the navigation bar, the browsing canvas and the detailed-information/user-space panel. Through the navigation bar, user controls the behavior of the browser, including open/close tracks and location/sequence query. The browsing canvas provides a map-like navigation function for user to browse the genomic features interactively. In addition, detailed annotation information and various user-generated contents could be displayed in the detailed-information/user-space panel.

The genome browser interface of ABrowse is divided into three parts: the main browsing canvas, the navigation control bar and the detailed-information/user-space panel (Figure 2). The displaying tracks in the main browsing canvas are listed in the "Current Tracks" tab of the detailed-information/user-space panel, where user can freely reorder the relative position of tracks by simple drag and drop. After clicking the cross mark, the selected track will be closed for customized browsing. Moreover, user can also place the target track on the top of the main browsing canvas by clicking the listed track name.

The annotation entries shown in the main browsing canvas are all clickable, and their corresponding detailed information can be listed in the "Entry Detail" tab of the detailed-information/user-space panel. For users with low resolution screen, ABrowse allows them to popup the detailed information panel in an independent in-page window.

To promote comparative analysis, ABrowse supports users to inspect several genomic regions simultaneously in multiple independent in-page windows with different views, inspiring novel discoveries among different species (Additional file 1, Figure S2).

### Open Data Access

With the increase of online resources and analysis tools, interactions among individual applications become more and more important, making open data accessibility by external systems a mandatory function for a genome browser. Multiple approaches are provided for external applications to access data in ABrowse, including online data submission to external analysis platforms for end users, as well as machine-oriented data retrieval protocol for developers.

For end users, ABrowse provides a one-stop seamless visualization-query-analysis service, supporting several approaches to submit selected sequences, annotations and comments to external bioinformatic platforms for further analysis, e.g., Galaxy [25,26] and WebLab [27] (Additional file 1, Figure S3). By simple clicks, various types of selected data from the built-in query system and BioMart can be transmitted transparently to external systems, avoiding manual downloading and uploading.

**Figure 3:**
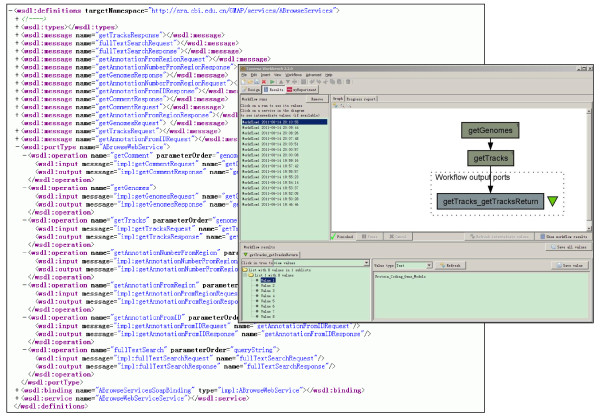
**Web Service interface and Taverna workflow support**. ABrowse web service is implemented with SOAP standard for data transporting, and it provides a WSDL file to specify the interaction interface with external applications. By supporting the standard web service, ABrowse provides programmatic access to the underlying database, helping users to integrate ABrowse into their applications. As shown in the figure, the web service is compatible with Taverna workbench of the myGrid project, helping to integrate several applications to perform data analysis as a workflow based on standard protocol.

For developers, ABrowse supports the standard SOAP-based web service interface to retrieve bulk data remotely, providing underlying machine-oriented protocol for other applications to access data openly. The web service is also compatible with Taverna workflow platform [28,29] and other web service supported systems (Figure 3). In order to assist developers to quickly deploy the web service applications, both the detailed WSDL file for interface description and client demo examples are provided online.

**Figure 4:**
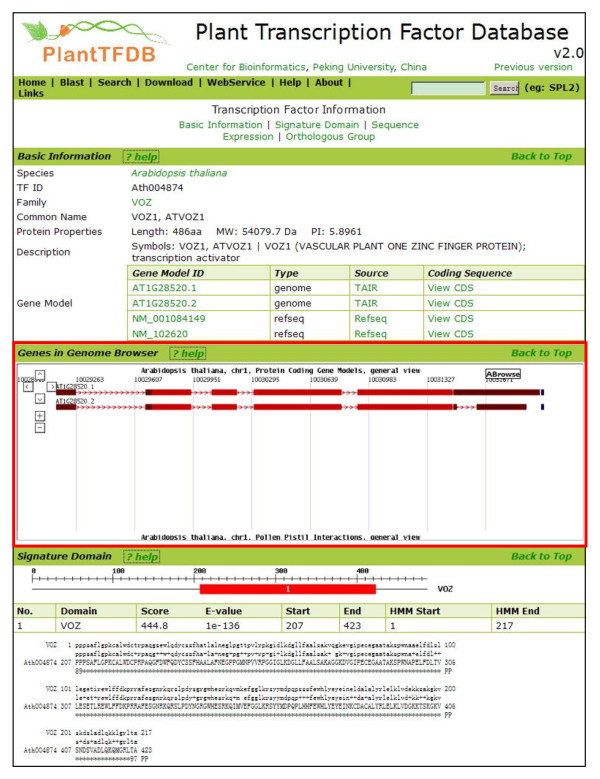
**Embed the genome browser canvas in a standalone web page**. The browsing canvas of ABrowse can be embedded into other web pages as a widget easily. As shown in the figure, the *Arabidopsis *genome browser canvas is embedded into a demo page for *Arabidopsis *transcription factor from PlantTFDB http://arabidopsis.cbi.edu.cn/web_plugin/ara.tf.html, promoting quick data sharing among isolated systems in graphical view.

Besides the online submission methods for end users and machine-oriented data retrieval protocol for developers, the entire genome browser canvas can be easily embedded into standalone web pages as a widget, promoting quick data sharing among isolated systems in graphical view (Figure 4).

### Collaborative Work Support

Collaborations among researchers from different organizations are becoming crucial for research success. Web 2.0 brings new ideas to promote users to establish a social, collective and collaborative platform for data creation, sharing and integration [18]. Thus, ABrowse provides rich support for user-generated contents, efficiently promoting information sharing among researchers worldwide.

Registered users can attach comments and stars for a track as community feedback, similar to the book review mechanism in Amazon. Users can also add rich text comments for existing annotation entries instantly as research notes (Additional file 1, Figure S4). In addition to writing comments on the existing annotations, ABrowse provides "My Instant Note" track for every registered user, supporting user to select any genomic region on-the-fly by clicking-and-dragging, and attach comment for it interactively. Furthermore, users can easily upload their own annotations to the browser from the web interface, and manage them by clicking the "My Tracks" tab in the detailed-information/user-space panel. When users find an interesting discovery and want to make a record, they can store current browsing status as a landmark, and then revert to the saved status at any time.

To promote collaborative work, ABrowse supports users to publish or share their comments, annotations and landmarks among colleagues. On the other hand, users can also keep their contributed data private as personal research notes. Furthermore, a query system for user comment is provided to conveniently search comments on specified track or item, and the retrieved comments can also be accessed by external applications transparently.

### Setup and Customization

The ABrowse framework is easy to install, highly customizable and configurable. Administrators and developers can customize and tune multiple visualizing elements to easily meet their own requirements.

It is easy for the site administrator to setup a new genome browser instance and import annotation without any programming. ABrowse supports data loading from both command line and web page with standard formats, such as GFF, SAM, BED, WIG, Microarray defined format, and the ABrowse defined format (Additional file 1, Figure S5). In order to load data automatically, a set of utilities are provided for various data importing, concealing all the intermediary steps for users. In addition, ABrowse can also be built based on existing databases by specification of corresponding SQL for data query in the configure file, providing loose coupling design between database layer and logic processing layer. To customize the "Entry Detail" page in the detailed-information/user-space panel, site administrators can add their own rendering JSP pages for tracks to meet specific display requirements.

As a general-purpose framework, ABrowse provides several easy-to-integrate interfaces for developers. Besides pre-defined visualization graphs and color schema, developers can easily integrate new elements into the framework by adding new drawing strategies. It is also easy to submit data from ABrowse to other platforms for further analysis. External platforms could implement the standard interface provided by ABrowse to accept data from an ABrowse instance transparently.

A live demo for the *Arabidopsis thaliana *genome http://arabidopsis.cbi.edu.cn/ has been built as a demonstration of all the features of ABrowse. And the detailed descriptions for installation, configuration and development interfaces are provided in the "Administrator Guide" and "Developer Guide" pages for different users to deploy and customize their own genome browser instances on the basic ABrowse framework.

### Usage and Future Plans

Currently, the ABrowse framework has been used in several internal and external projects. We have built Rice-Map http://www.ricemap.org/[30] based on a customized version of ABrowse as a new generation rice genome browser. Moreover, ABrowse framework has been used by several research institutions as their local genome browsers, including the Institute of Molecular Medicine of Peking University for the RhesusBase project, the Chinese Academy of Fishery Sciences for the Carp genome project, as well as the Institute of Vegetables and Flowers of Chinese Academy of Agricultural Sciences for the Brassica genome project.

ABrowse is an open source genome browser framework for not only end users, but also data providers and developers. Powered by cutting-edge technologies, ABrowse provides a rather comprehensive set of features as a modern next-generation genome browser framework (Additional file 2, Table S1). By supporting map-like navigating experience through AJAX, ABrowse offers a highly interactive user interface with much improved user experience than classical page-based layout. To promote collaboration, ABrowse provides dedicated personal data space for all registered users to keep and share their own annotations and working notes with colleagues. In addition to rich interface, ABrowse also built in with a powerful query system for both pre-computed and user-generated annotation, including text-oriented full text search and sequence-oriented query. Using a BioMart-compatible schema, ABrowse enables site administrators to take full advantages of the well-designed BioMart engine. Moreover, ABrowse provides native SOAP-based web service API, allowing easy integration with various existing analysis tools. In the future, we shall continue to maintain and develop ABrowse through following new technologies, and collaborating with academic and industrial partners.

## Conclusions

In response to the increasing demands for a general-purpose genome browser framework, we have developed a next-generation genome browser framework ABrowse which provides interactive browsing experience, open data access and collaborative work support. Taking advantage of the new computing technologies, ABrowse provides highly flexible and configurable interfaces, supporting administrators and developers to easily customize and tune visualizing elements.

## Availability and Requirements

ABrowse is an open genome browser framework, and the source codes are released under GNU Lesser General Public License v3.0, publicly available for free downloading at http://www.abrowse.org/. To setup an ABrowse instance, the pre-requested software Tomcat, MySQL and Java runtime environment are needed.

## Authors' contributions

**LK **and **JW **conceived and carried out the research, and drafted the paper; **SQZ **wrote some programs and jointly helped to revise the manuscript with **XCG**; **GG **and **JCL **conducted the research and revised the manuscript. All authors have read and approved the manuscript.

## Supplementary Material

Additional file 1 **ABrowse supplementary figures**. Click here for file

Additional file 2 **ABrowse supplementary feature table**. Click here for file
