# Supplementary material for: ABrowse - a customizable next-generation genome browser framework
Source: BMC Bioinformatics. 2012 Jan 5;13:2. doi: 10.1186/1471-2105-13-2 (PMC3265404; doi:10.1186/1471-2105-13-2)
Supplement: Additional file 1 — ABrowse supplementary figures. [file 1471-2105-13-2-S1.PPTX]

## Slide 1
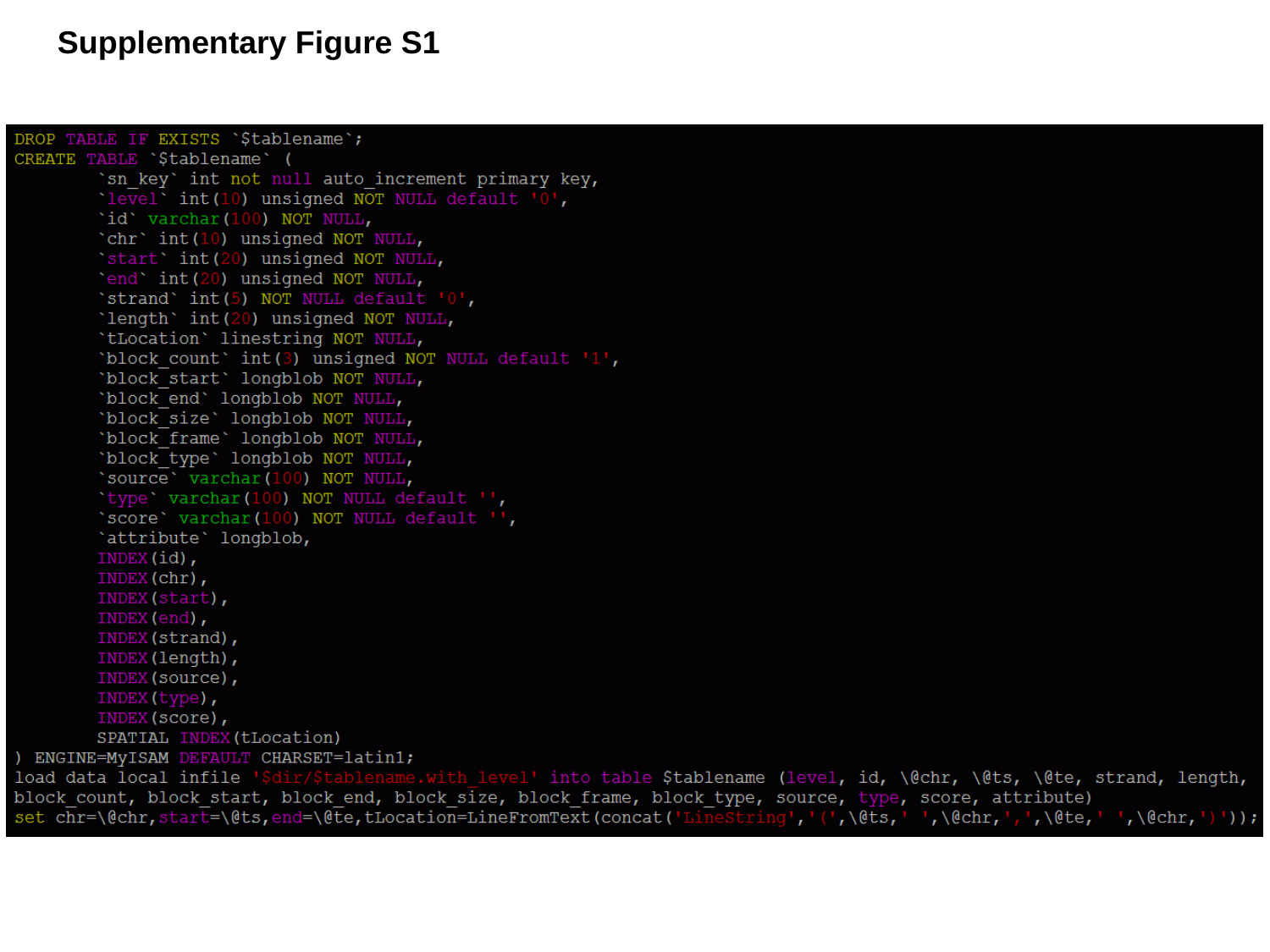

Supplementary Figure S1

## Slide 2
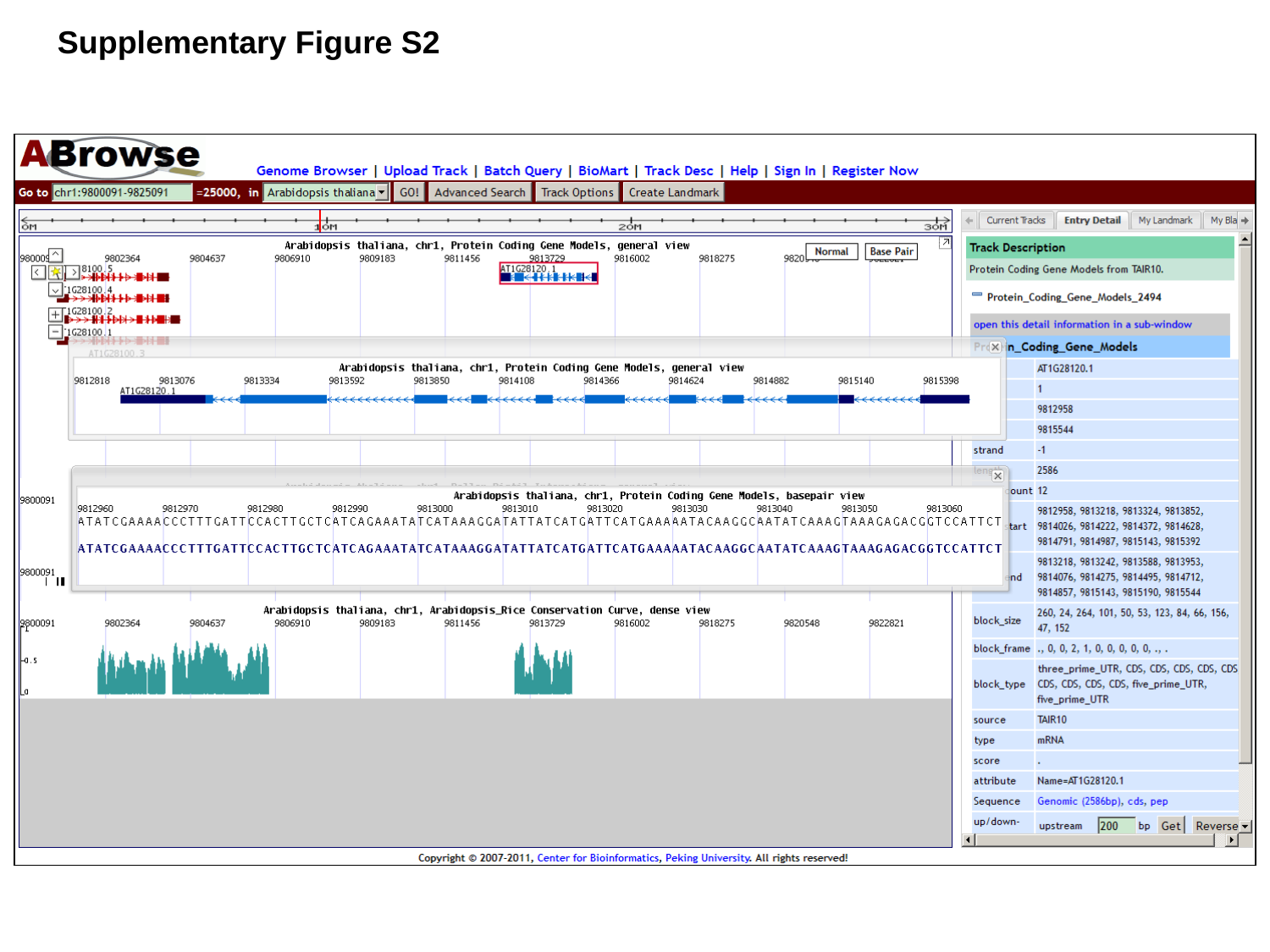

Supplementary Figure S2

## Slide 3
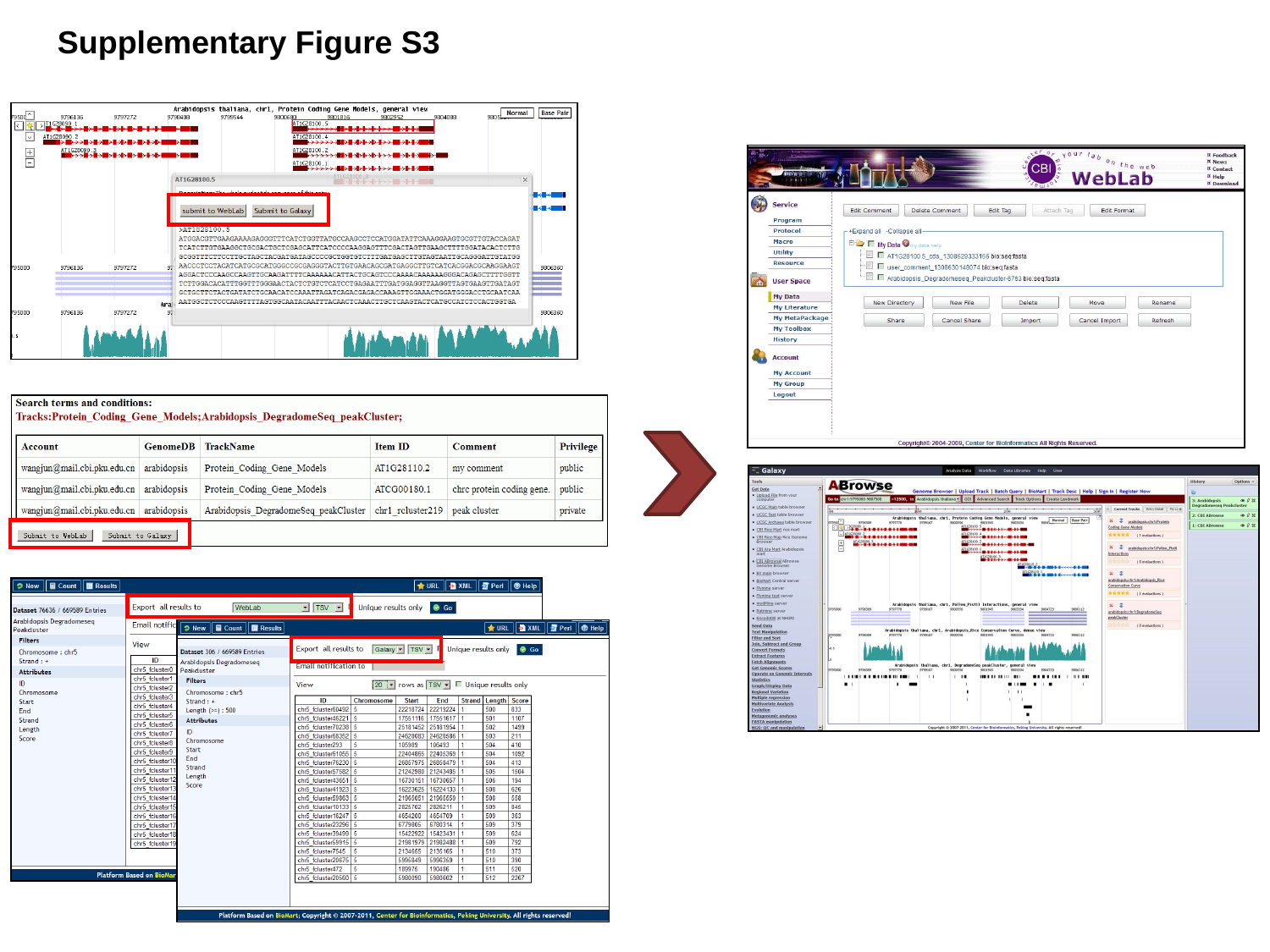

Supplementary Figure S3

## Slide 4
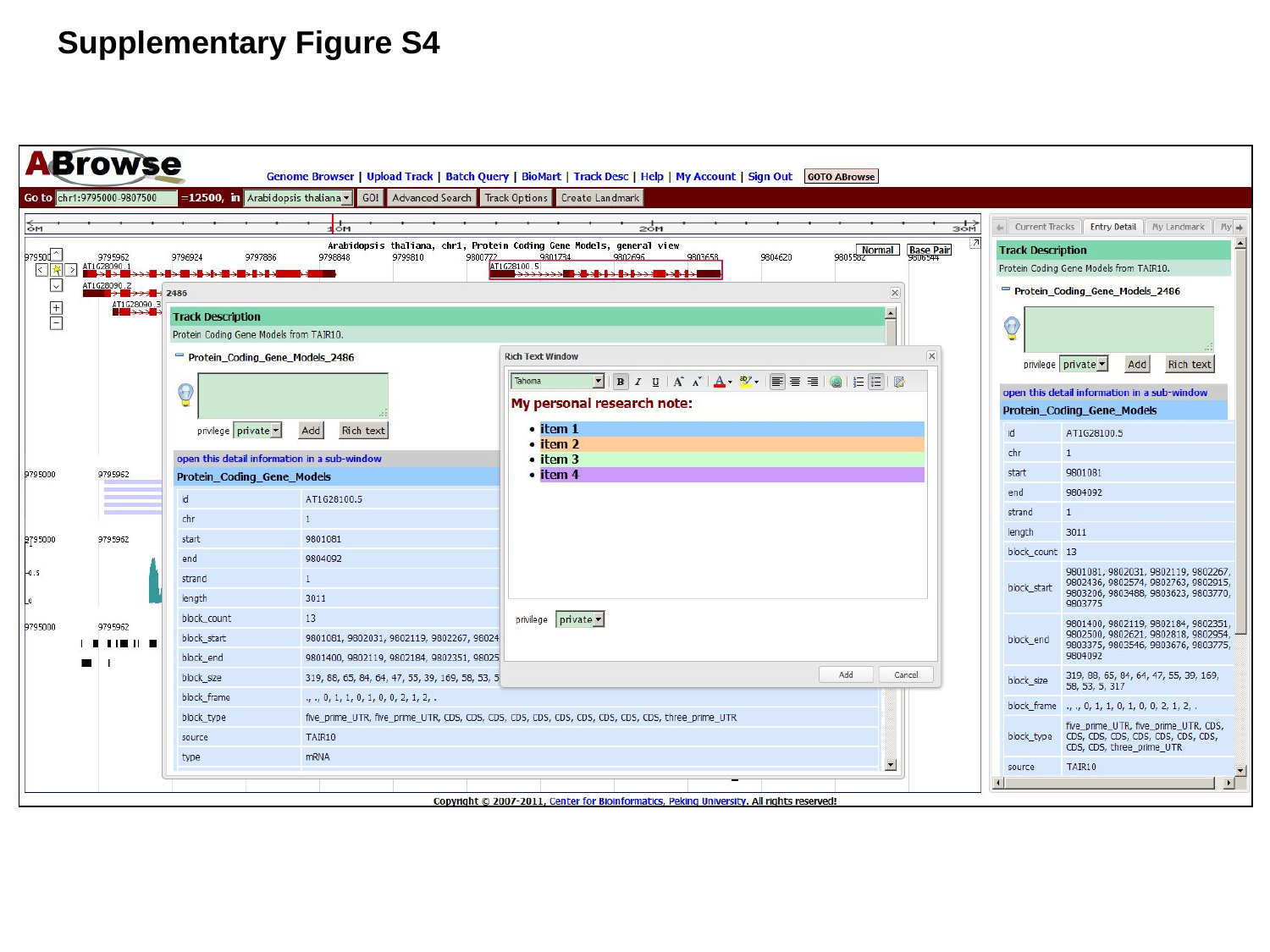

Supplementary Figure S4

## Slide 5
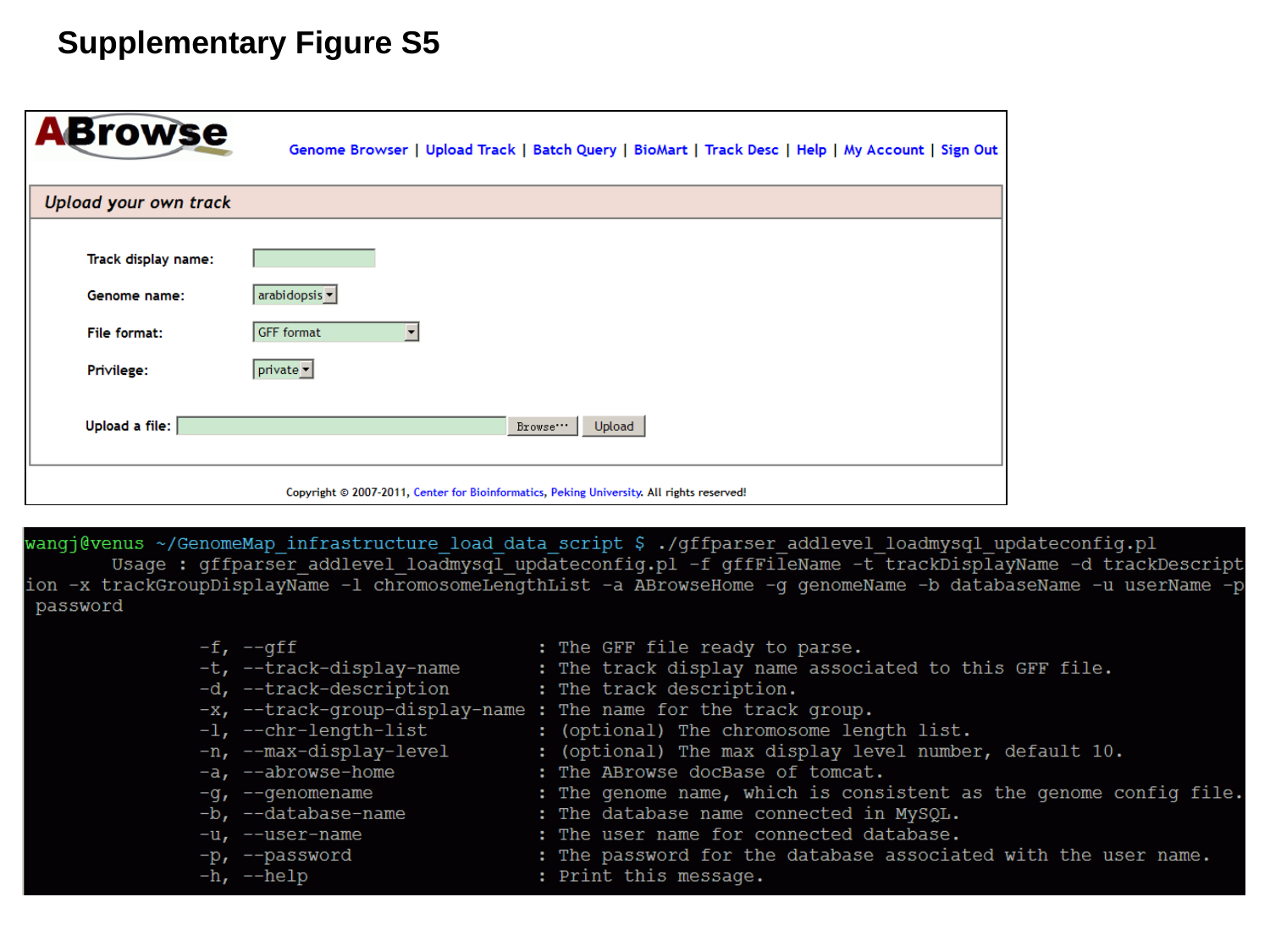

Supplementary Figure S5

## Slide 6
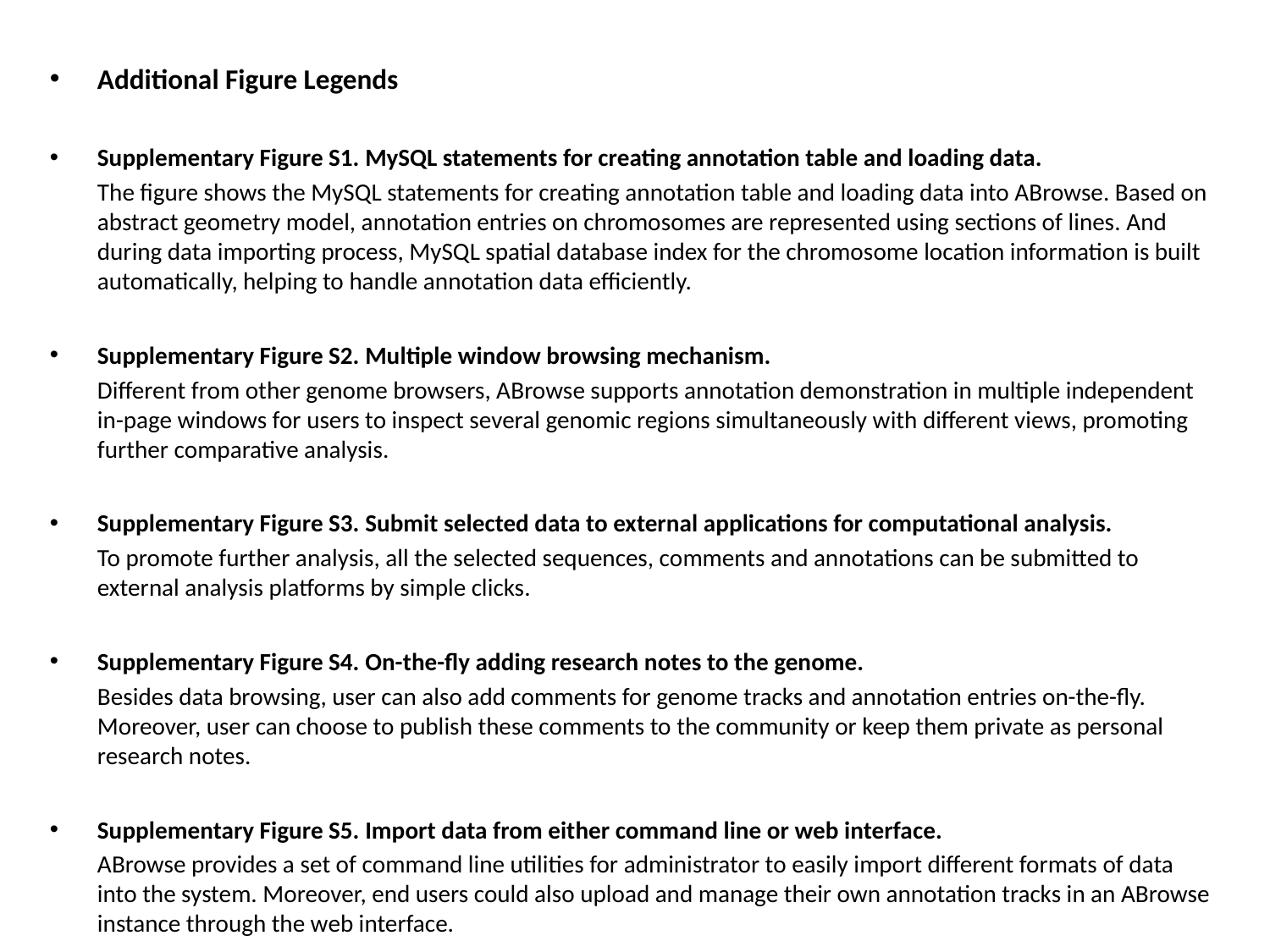

Additional Figure Legends
Supplementary Figure S1. MySQL statements for creating annotation table and loading data.
	The figure shows the MySQL statements for creating annotation table and loading data into ABrowse. Based on abstract geometry model, annotation entries on chromosomes are represented using sections of lines. And during data importing process, MySQL spatial database index for the chromosome location information is built automatically, helping to handle annotation data efficiently.
Supplementary Figure S2. Multiple window browsing mechanism.
	Different from other genome browsers, ABrowse supports annotation demonstration in multiple independent in-page windows for users to inspect several genomic regions simultaneously with different views, promoting further comparative analysis.
Supplementary Figure S3. Submit selected data to external applications for computational analysis.
	To promote further analysis, all the selected sequences, comments and annotations can be submitted to external analysis platforms by simple clicks.
Supplementary Figure S4. On-the-fly adding research notes to the genome.
	Besides data browsing, user can also add comments for genome tracks and annotation entries on-the-fly. Moreover, user can choose to publish these comments to the community or keep them private as personal research notes.
Supplementary Figure S5. Import data from either command line or web interface.
	ABrowse provides a set of command line utilities for administrator to easily import different formats of data into the system. Moreover, end users could also upload and manage their own annotation tracks in an ABrowse instance through the web interface.
