# Supplementary material for: ABrowse - a customizable next-generation genome browser framework
Source: BMC Bioinformatics. 2012 Jan 5;13:2. doi: 10.1186/1471-2105-13-2 (PMC3265404; doi:10.1186/1471-2105-13-2)
Supplement: Additional file 2 — ABrowse supplementary feature table. [file 1471-2105-13-2-S2.DOC]

**Supplementary Table S1. ABrowse feature table, with comparison to other genome browser frameworks.**

| **Features** | | **ABrowse** | **GBrowse2** | **JBrowse** | **Anno-J** | **Genome Projector** | **UCSC** | **Ensembl** |
| --- | --- | --- | --- | --- | --- | --- | --- | --- |
| **User interface** | Map-like navigation, i.e. smoothly dragging zooming, and scrolling along whole genome. | **√** | **×^*^** | **√** | **√** | **√** | **×** | **×** |
|  | Multiple in-page windows | **√** | **×** | **×** | **×** | **×** | **×** | **×** |
|  | Browsing history/Bookmark | **√** | **√** | **×** | **×** | **×** | **√** | **√** |
|  | Track reorder | **√** | **√** | **√** | **√** | **×** | **√** | **√** |
| **Query system** | Full text search | **√** | **√** | **×** | **√** | **√** | **√** | **√** |
|  | Advanced search for pre-compiled annotation | **√** | **×** | **×** | **×** | **×** | **√** | **×** |
|  | Advanced search for user-generated content | **√** | **×** | **×** | **×** | **×** | **×** | **×** |
|  | BioMart-compatible | **√** | **×** | **×** | **×** | **×** | **×** | **√** |
|  | Sequence-based search | **√** | **√** | **×** | **×** | **√** | **√** | **√** |
| **Submit data to external platforms** | Pre-compiled annotation and sequence | **√** | **×** | **×** | **×** | **×** | **√** | **×** |
|  | User-generated content | **√** | **×** | **×** | **×** | **×** | **×** | **×** |
| **Personalized workbench** | Add comments for annotation entries | **√** | **×** | **×** | **×** | **×** | **×** | **√** |
|  | Make evaluation for tracks | **√** | **×** | **×** | **×** | **×** | **×** | **×** |
|  | Sequence-based search history | **√** | **×** | **×** | **×** | **×** | **×** | **×** |
|  | Customized user tracks | **√** | **√** | **√** | **×** | **×** | **√** | **√** |
|  | Share user space contents | **√** | **√** | **√** | **×** | **√** | **√** | **√** |
| **Development** | Web Service support | Native | Through BioDAS | Through Amazon | **×** | **×** | Through BioDAS | Through BioMart |
|  | Embedded as widget within user-specified page | **√** | **√** | **√** | **×** | **×** | **×** | **×** |

* Supporting local smooth browsing mode (i.e. dragging detail tracks left and right without having to load more data from the server within a limited region specified by “details multiplier” property) since version 2.20, see <http://cpan.uwinnipeg.ca/htdocs/GBrowse/Changes.html> for details.
